# Updates on Old and Weary Haematopoiesis

**DOI:** 10.3390/ijms19092567

**Published:** 2018-08-29

**Authors:** Joanna Konieczny, Lorena Arranz

**Affiliations:** 1 Stem Cell Aging and Cancer Research Group, Department of Medical Biology, Faculty of Health Sciences, UiT, The Arctic University of Norway, 9019 Tromsø, Norway; jko029@post.uit.no; 2 Department of Hematology, University Hospital of North Norway, 9019 Tromsø, Norway; 3 Young Associate Investigator, Norwegian Center for Molecular Medicine (NCMM), 0349 Oslo, Norway

**Keywords:** haematopoiesis, ageing, clonal haematopoiesis, leukaemia, bone marrow, haematopoietic stem cell niche, inflammageing

## Abstract

Blood formation, or haematopoiesis, originates from haematopoietic stem cells (HSCs), whose functions and maintenance are regulated in both cell- and cell non-autonomous ways. The surroundings of HSCs in the bone marrow create a specific niche or microenvironment where HSCs nest that allows them to retain their unique characteristics and respond rapidly to external stimuli. Ageing is accompanied by reduced regenerative capacity of the organism affecting all systems, due to the progressive decline of stem cell functions. This includes blood and HSCs, which contributes to age-related haematological disorders, anaemia, and immunosenescence, among others. Furthermore, chronological ageing is characterised by myeloid and platelet HSC skewing, inflammageing, and expanded clonal haematopoiesis, which may be the result of the accumulation of preleukaemic lesions in HSCs. Intriguingly, haematological malignancies such as acute myeloid leukaemia have a high incidence among elderly patients, yet not all individuals with clonal haematopoiesis develop leukaemias. Here, we discuss recent work on these aspects, their potential underlying molecular mechanisms, and the first cues linking age-related changes in the HSC niche to poor HSC maintenance. Future work is needed for a better understanding of haematopoiesis during ageing. This field may open new avenues for HSC rejuvenation and therapeutic strategies in the elderly.

## 1. Introduction

Haematopoiesis is the process of the generation of all differentiated blood cells in the organism, including red blood cells, platelets, innate immune cells, and lymphocytes; all found to fade in functionality in aged individuals. Haematopoiesis is carried out by a rare population of haematopoietic stem cells (HSCs), which in adults, reside mainly in the bone marrow. There, they either remain dormant, i.e., in a quiescent state, or undergo proliferation and differentiation, depending on their cell-intrinsic transcriptional programs and the external cues from the surroundings. In both humans and mice, advances in highly purified or single-cell transcriptomics and functional techniques challenge the past concept of cellular hierarchy in the haematopoietic system, where HSCs were thought to differentiate into a series of multilineage progenitors, culminating in unilineage progenitors that give rise to the variety of differentiated cells. Rather, adult HSCs seem to be a heterogeneous subset of mainly multipotent and unipotent progenitors affiliated to specific lineages, and the ratio of their skewing shifts when homeostasis is perturbed [1,2,3]. HSC maintenance relies on the support from the microenvironment or niche, which tightly controls their function, fate, and numbers [4]. The HSC niche, a concept cued by Schofield already in 1978 [5], is necessary to preserve the self-renewing potential of HSCs [4], which ensures the provision of newly differentiated blood cells whilst maintaining the HSC pool itself [6]. Extensive research on HSC niches composition shows that they are closely related to the vasculature in the bone marrow, with mainly endothelial, perivascular, and mesenchymal stromal cells secreting factors that support HSC maintenance [7]. In this scenario, the effects of ageing on haematopoiesis may be the result of age-related alterations in all blood cell subsets, including HSCs and progenitors, as well as in the HSC niche.

## 2. HSC Ageing and Myeloid/Platelet Skewing

In adult stem cells, ageing is accompanied by exhaustion of their self-renewing potential: their main feature [8]. Interestingly, in mice, the number of phenotypically defined HSCs can increase up to tenfold with ageing [9]. In contrast, their functionality in terms of self-renewal and repopulating ability is remarkably reduced [9]. Use of cellular barcoding combined with multiplex deep sequencing demonstrated that clonal HSC composition in old mice shows increased variability of clones derived from a single stem cell with smaller size per clone, when compared to young mice [10]. Competitive transplantation of these HSCs proved that young HSCs perform better, with three-fold higher yield of mature granulocytes and lymphocytes [11]. Furthermore, age-related defective HSCs seem to be able to differentiate into the myeloid lineage, but are incapable of the balanced generation of lymphocytes following transplantation [11]. 

**Figure 1:**
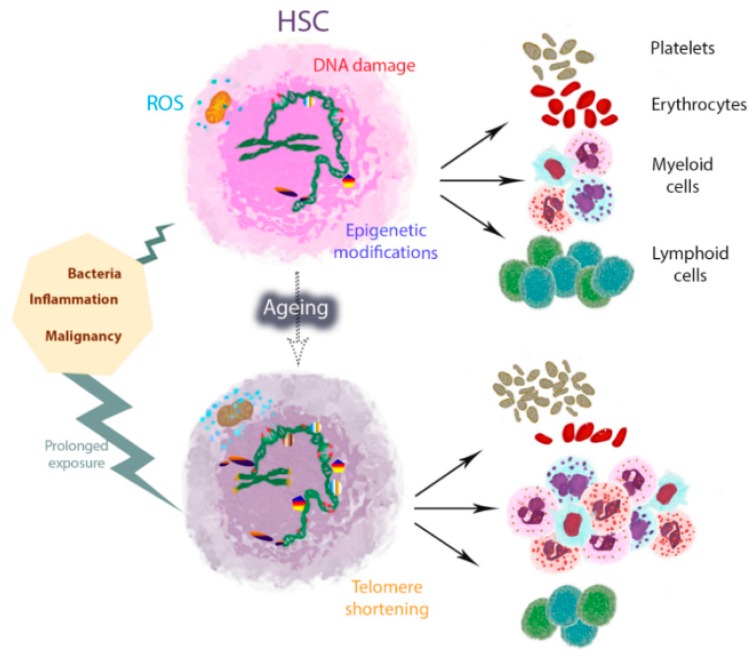
Model of haematopoietic stem cell (HSC) myeloid and platelet skewing with ageing in mice. One of the typical characteristics of HSC ageing is myeloid and platelet HSC skewing, which is accompanied by profound changes in the epigenetic landscape and gene expression profiles. HSC activation in response to external stimuli such as infections and inflammation elicits HSC differentiation and myeloid skewing, aimed at mediating rapid myeloid cell recovery at the expense of their self-renewal capacity. HSCs shift from quiescence to more cycling states, with increased reactive oxygen species (ROS) levels and DNA damage. Prolonged exposure during life may potentially cause the accumulation and aggravation of changes, including telomere shortening, ultimately reducing HSC survival and differentiation potential. The course of lifetime is represented by a dotted arrow.

Thus, HSC defects are reflected in insufficiencies in their progeny of differentiated cells and contribute to poorer systemic performance of the haematopoietic system, i.e., immunosenescence [12], in the elderly, particularly adaptive immunity [13,14] (Figure 1). Concomitant with HSC expansion, ageing is accompanied by an early and progressive loss of lymphoid-primed multipotent progenitors that show increased cycling, as well as reduced lymphoid priming and differentiation potential [15]. In contrast, myelopoiesis was reported to be relatively unaffected by ageing, as numbers of common myeloid progenitors and their progeny remain unchanged or increased in old mice [16,17]. However, more recent data suggest that defects also extend to aged myeloid progenitors [18], and include increased cycling and reduced survival and repopulating potential, similarly to HSCs [18,19]. Then, defects in progenitors may also result in altered functionality in their progeny of differentiated myeloid cells. This may contribute to the compromised innate immunity reported during ageing, by means of the diminished function of neutrophils [20], macrophages [21], and dendritic cells [22], adding up to their age-related cell-intrinsic defects [23]. 

Myeloid skewing results from the downregulation of lymphoid and upregulation of myeloid differentiation genes in aged HSCs [17], with disruption in epigenetic profiles [9,24,25] (Figure 1). Thus, myeloid skewing is performed at the expense of the lymphoid lineage. Actually, one contributing factor to the prevalence of myeloid-dominant HSCs in old mice is that old myeloid-skewed HSCs are generated from additional sources to young myeloid-primed HSCs [26]. It is currently unknown why the composition of the HSC compartment shifts during ageing. Several contributing processes have been postulated, including slower turnover and longer survival of myeloid-primed HSCs compared to other HSC subsets, or higher self-renewal capacity leading to clonal dominance [27]. Because lymphocytes have a longer life span than myeloid cells, it is reasonable to hypothesise that infections and exposure to microbes will primarily influence myeloid-biased HSCs, aiming at fast myeloid cell recovery. Future studies should test the validity of this exciting idea.

Myeloid-primed HSCs may be distinguished by the expression of one selective marker, CD41: an integrin characteristic of platelets, which increases with age [28]. This marker is also used to discriminate stem-like megakaryocyte-committed progenitors contained within the HSC pool during inflammation [29]. In this regard, a myeloid and platelet-primed HSC subset was additionally defined recently by the expression of von Willebrand factor (vWF) [1]. Considering this feature plus functional platelet bias at the single-cell level, platelet-primed HSCs were reported to increase fiftyfold during ageing in mice [30]. Interestingly, a high proportion of aged HSCs almost exclusively produce platelets, and taking this into consideration, no age-related reduction in the frequency of HSCs able to engraft after transplantation was found. Moreover, the platelet gene expression programme may contribute to the lymphoid lineage suppression, as depletion of the first leads to an increase of the latter [30]. Future work is required for a better understanding of the relationship between myeloid and platelet HSC skewing during ageing.

At the molecular level, DNA damage and telomere shortening seem to be major mechanisms underlying the age-related decrease in the functionality and durability of HSCs [31] (Figure 1). Interestingly, HSCs and progenitors are protected from DNA damage, as they do not experience an increase in mutation rate upon irradiation-induced DNA damage repair and they preferentially undergo apoptosis rather than defective repair upon chronic DNA double-strand breaks [32]. DNA damage triggers activation of cell-intrinsic checkpoints such as p53 and retinoblastoma, downstream targets including p21 and sestrins, and upstream regulators such as p16INK4a and p19ARF. Induction of cell-intrinsic checkpoints is aimed at clearing the damaged cells, preventing leukaemic transformation, but it may also impair HSC pool maintenance and fitness of the haematopoietic system through prolonged action [33]. Interestingly, transgenic mice with increased p53 function display several premature ageing phenotypes, including defects in HSC proliferative capacity and regeneration activity [34]. However, these HSCs appear younger at the molecular level with younger expression patterns in a variety of gene ontology categories, including response to DNA damage, protein folding, RNA processing, or chromatin modification, but not the inflammatory response, when compared to their wild-type and p53+/− counterparts [9]. This suggests that disruption of the cell cycle in HSCs results in the partial uncoupling of tissue and HSC ageing [9]. It also highlights the key role of the inflammatory response in age-related HSC functional impairment.

Besides, telomeres are fragile sites in the genome and thus sensitive to DNA damage [33]. HSCs lose telomeric DNA with each division [35] and this hinders their proliferative potential over time [36]. Protective mechanisms, such as the protection of telomeres-1 (Pot1a) protein expression, diminish with ageing in mice [37]. Furthermore, telomerase confers extended replicative capacity to HSCs and its deficiency impairs HSC function, particularly under stress [31,38], leading to the exhaustion of functional HSCs in secondary recipients in serial transplantation [31]. Recently, telomerase activity was found crucial for erythropoiesis, and importantly, the deleterious effects of deficient telomerase activity on HSC and progenitor cell proliferation, DNA damage response, red cell production, and haemoglobin levels were reported as reversible through Cre-induced expression of the gene [39]. This suggests that novel strategies aiming at restoring telomerase function with ageing may have an important implication in the clinical setting to rejuvenate HSC function.

DNA damage accumulation is intimately related to increased reactive oxygen species (ROS) levels [40]. In fact, HSCs reside in hypoxic bone marrow niches, which maintain their long-term self-renewal by mechanisms such as limiting their ROS production [41]. This is performed through adaptation of their metabolism to maintain a high glycolysis rate, whereas during activation of proliferation and differentiation, HSCs depend more on oxidative phosphorylation to meet their energy requirements [41]. Stressors, such as infections or chronic blood loss, shift HSCs from the quiescent to cycling state, which consequently leads to increased ROS levels and DNA damage (Figure 1). This may be the reason for the premature bone marrow failure present in Fanconi anaemia and may as well contribute to normal HSC ageing [42]. Interestingly, increased ROS induces p38 mitogen-activated protein kinase (MAPK) signalling, and several strategies targeting this pathway have successfully protected HSCs against loss of self-renewal, including prolonged treatment with antioxidant or inhibitors of p38 MAPK [43,44].

## 3. Inflammageing and Its Relation to HSC Ageing

Inflammageing is the characteristic process of chronic inflammation that has been described in aged individuals [45], with an increase of inflammatory cytokine levels that correlate with morbidity and age-related diseases [46]. Inflammation is a natural response of the organism towards pathogens, tissue damage, and other endogenous stimuli such as tumour cells. The HSC compartment is tightly connected to inflammatory processes, as a producer of innate immune cells. Furthermore, HSCs express pattern recognition receptors required for the identification of dangers, and a variety of cytokines and their receptors [47,48]. Activation of these signalling pathways elicits HSC differentiation and myeloid skewing [48,49], aimed at mediating rapid myeloid cell recovery. However, when not finely regulated, they may cause HSC exhaustion [49] (Figure 1). Whether inflammageing contributes to the age-related defects observed in HSCs and/or HSCs actively participate in the process is currently unclear and should be a subject of future research.

In elderly individuals, a variety of factors such as interleukin 6, interleukin 1 receptor antagonist, interleukin 18, fibrinogen, and C reactive protein all increase significantly, with interleukin 6 soluble receptor increase observed only in men [46]. In mice, cytokines such as interleukin 1 beta, interleukin 6, interferon gamma, and tumour necrosis factor alpha are significantly increased during ageing. Interestingly, their levels are reduced in healthy long-lived individuals as compared to non-selected old mice [50], as further indication of their potential damaging effect. Several factors may contribute to the differences seen between humans and mice, such as sample type (serum and peritoneal suspension, respectively) and detection methods of different sensitivity (colorimetric ELISA and multiplexed fluorometric immunoassay, respectively) [46,50]. The case of interleukin 1 beta is of particular interest, as this is one of the cytokines produced by HSCs [51] that induces myeloid differentiation and limits self-renewal in mice [49]. Patients with mutations in the nucleotide-binding domain leucine-rich repeat containing protein 3 gene, which controls the caspase 1 activity in charge of interleukin 1 beta activation, have high levels of interleukin 6 and C reactive protein, among others [52]. The levels of these factors decrease rapidly upon blockade of the interleukin 1 receptor, suggesting that interleukin 1 beta contributes to the elevation of these markers in this inflammatory disease and may also contribute to inflammageing [52]. In fact, the increase in interleukin 1 receptor antagonist seen in elderly humans may reflect a homeostatic effect of the organism attempting to control inflammation through interleukin 1 beta targeting. Future work should test this exciting hypothesis. 

In addition, among cytokines produced by HSCs and progenitors, interleukin 6 seems to be important as a regulator of their proliferation and myeloid differentiation in a paracrine manner, and as a driver of myelopoiesis both in vitro and in vivo in neutropenic mice after chemotherapy or bone marrow transplant [48]. Besides, tumour necrosis factor added to cycling human HSCs both in vitro and in vivo compromises their ability to reconstitute immunodeficient mice and long-term cultures. This effect is mediated by the tumour necrosis factor receptor p55, which seems to promote HSC differentiation [53]. Interestingly, induction of the interferon response in mice, through polyinosinic/polycytidylic acid injection that mimics acute inflammation, leads to a fast decline in platelet numbers, which are restored within a few days. This emergency megakaryopoiesis occurs in response to increased interferons, and a stem-like megakaryocyte-committed progenitor subset that expresses CD41 and is contained within the HSC pool is responsible for it [29]. Taken together, these data suggest that the high levels of inflammatory cytokines seen in the elderly may indeed contribute importantly to the HSC skewing towards myeloid lineages and platelets during ageing. 

Chronic inflammation also plays a role in age-related diseases, particularly haematological malignancies. An epidemiological study based in Sweden revealed that history of any infectious disease was associated with a 1.3-fold significantly increased risk of acute myeloid leukaemia (AML) or myelodysplastic syndrome, even when infection had occurred years before onset [54]. In addition, infection seems to be a causal factor in childhood acute lymphoblastic leukaemia [55,56]. Chronic inflammation and autoimmune diseases have also been linked with increased risk of malignant lymphomas in adults [57]. In patients with myeloproliferative neoplasms, chronic inflammation has been evidenced as a potential initiating event and driver of clonal expansion that predisposes to second cancer development [58,59,60]. In particular, enhanced interleukin 1 beta signalling is a common event in patients with haematological malignancies, and evidence obtained in preclinical models shows its pathogenic role and therapeutic potential in AML, myeloproliferative neoplasms, and juvenile myelomonocytic leukaemia, among others [61]. Patients with myelodysplastic syndromes show overexpression of tumour necrosis factor alpha and, in some cases, interferon gamma, which have been suggested to contribute to the disruption of haematopoiesis in these diseases [62]. In chronic myeloid leukaemia, mutated cells transform normal HSCs and progenitors into abnormal cells that resemble their malignant counterparts through IL-6 secretion [63]. Thus, targeting inflammation may have clinical implications to improve the treatment and/or prevent the onset of age-related haematological malignancies in elderly patients.

## 4. Ageing of the HSC Niche

**Figure 2:**
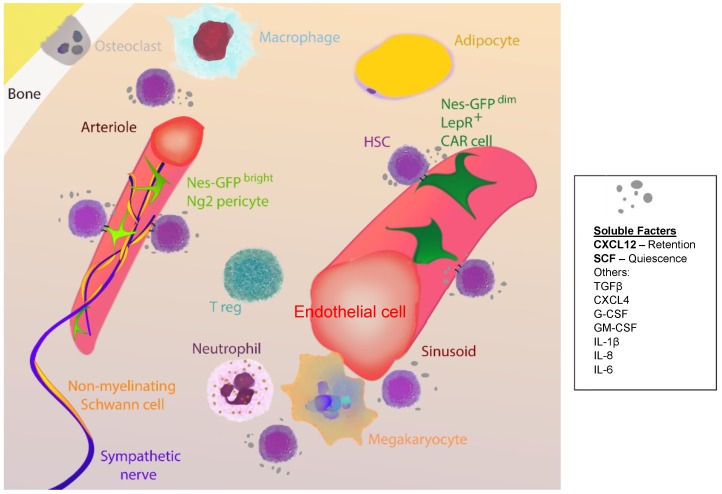
Major players in bone marrow haematopoietic stem cell (HSC) niches. HSC niches in the bone marrow are complex and closely related to the vasculature. The majority of HSCs localise near sinusoids associated with endothelial cells and leptin receptor (LepR)-expressing mesenchymal stromal cells, which partially overlap with CXC-chemokine ligand 12 (CXCL12)-abundant reticular (CAR) cells and Nestin (Nes)-GPF^dim^ cells. A fraction of HSCs localise adjacent to small-diameter arterioles, adjacent to neural/glial antigen (Ng)2 pericytes that partially overlap with Nes-GFP^bright^ cells. Other cell subsets that regulate HSC function include sympathetic fibres, non-myelinating Schwann cells, adipocytes, megakaryocytes, neutrophils, macrophages, T regulatory (reg) cells, and osteoclasts, either by direct or indirect mechanisms. Direct mechanisms include cell-to-cell contact and secretion of soluble factors, importantly stem cell factor (SCF) that regulates HSC quiescence and CXCL12 that promotes HSC retention. Other soluble factors that control HSC function are tumor growth factor beta (TGFβ), CXCL4, granulocyte colony-stimulating factor (G-CSF), granulocyte-macrophage colony-stimulating factor (GM-CSF), and several interleukins (IL), among others.

As previously mentioned, HSC survival and function relies on the support from the microenvironment or niche in the bone marrow [4]. Stem cell niches are complex and unique structures, yet they share many features that include cellular interactions, secreted factors, extracellular matrix, physical factors, metabolic conditions, and importantly, processes of scarring and inflammation [64]. Furthermore, bone marrow HSC niches are mainly perivascular, with mostly endothelial cells and mesenchymal stromal cells secreting factors that support HSCs, such as stem cell factor [7] (Figure 2).

An interesting study of the HSC rejuvenation ability of the young environment was presented by Mayack and colleagues [65]. They used the challenging parabiotic mouse model and surgical connection of the circulatory systems of two mice, young and old, which were compared to old or young parabionts as control groups. This model has certain limitations such as major surgery, poor animal welfare, and immune system alterations, but it provides valuable information. In heterochronic mice, the long-term HSC compartment of old mouse origin recovered to original youthful numbers and engraftment potential, with the restoration of youthful ratios of B lymphoid to myeloid cells, when exposed to the young environment. Similar results were obtained when coculturing HSCs with osteoblastic cells, defined as OPN^+^CD45^−^Ter119^−^, from young and old individuals, respectively. The insulin-like growth factor-1, a soluble factor associated with ageing that regulates differentiation, was found to participate in the ageing of the niche and age-related HSC dysfunction, and this may be targeted pharmacologically in aged osteoblastic niche cells to promote youthful HSC regulatory function [65].

The changes in the bone marrow niche of aged mice include reduced numbers of perivascular cells expressing the receptor for platelet-derived growth factor beta and neural/glial antigen 2, alpha smooth muscle actin-covered arteries, and endomucin-expressing capillaries [66] (Figure 2). In aged mice, enhancement of the Notch signalling pathway in endothelial cells increases CD31^hi^ capillaries and CD31^+^ arterioles, ephrin-B2^+^ endothelial cells, and platelet-derived growth factor beta-positive and neural/glial antigen 2-positive perivascular cells. Furthermore, niche-forming vessel improvements are followed by increased HSC numbers, but no changes in their functionality. This was explained on the basis of persistent HSC cell-autonomous alterations such as DNA damage [66]. Thus, although factors such as duration of the treatment and time of initiation should be considered, this suggests that niche-based rejuvenating strategies may have only partial efficiency to recover HSCs to a youthful state.

However, other authors have found that the Nestin-GFP^bright^ cell subset that partially overlaps with neural/glial antigen 2-positive arterioles expands, whereas Nestin-GFP^dim^ cells that partially overlap with leptin receptor-positive mesenchymal stromal cells seem to be unchanged [67,68] (Figure 2). In compact bones from old mice, the frequency of Nestin-GFP^+^ cells is significantly reduced, as it is their colony-forming capacity ex vivo [68]. Sympathetic nerves regulate HSC function, acting through adrenoreceptor beta 3 on Nestin-GFP^+^ mesenchymal stromal cells [69] (Figure 2), and surgical denervation of sciatic and femoral nerves leads to premature HSC ageing, as evidenced by increased proliferation and specific myeloid bias [68]. Strikingly, supplementation with adrenoreceptor beta 3 agonist to old mice significantly rejuvenated the in vivo function of aged HSCs, evidencing this pathway as a good therapeutic target [68]. Furthermore, damage of the bone marrow sympathetic nervous system has been linked to the progression of age-related haematological malignancies, including myeloproliferative neoplasms [51] and AML [70]. Strategies aimed at restoring the physiological control of the sympathetic nervous system over the malignant cells through in vivo therapy with adrenoreceptor beta 3 agonist were capable of blocking disease progression [51]. Thus, improvement of the adrenoreceptor beta 3 pathway may be relevant to both rejuvenate HSCs during ageing as well as to prevent onset and/or improve the treatment of age-related haematological malignancies.

In addition, a recent study showed that vWF-expressing HSCs are highly enriched in megakaryocytic niches [71] (Figure 2). Interestingly, the depletion of megakaryocytes selectively expands this subset of HSCs, whereas the depletion of neural/glial antigen 2 arteriolar cells, previously shown to maintain HSC quiescence [72], preferentially depletes lymphoid-biased HSCs. Megakaryocyte depletion further compromises vWF-expressing HSC function by impairing their long-term self-renewal capacity and eliminating their lineage bias after transplantation [71]. Then, future work should evaluate the role of the megakaryocytic niche in HSC skewing during ageing. 

## 5. Clonal Haematopoiesis and Age-Related Haematological Malignancies

Acute myeloid leukaemia is the most frequent acute haematopoietic malignancy in adults. Its incidence increases with age and mortality exceeds 90% when diagnosed after the age of 65 years [73]. To date, the most accepted model for AML development suggests the requirement of mutations in at least two genes that specifically confer a survival advantage to the HSC and impede its further differentiation [74]. This proposal is based on the fact that oncogenes that confer a survival advantage to the HSC and are frequently mutated in human AML, such as RAS and FLT3, are only capable of inducing myeloproliferative neoplasms in mouse models, but not the transition to AML [75,76,77]. This indicates that other factors must participate in leukaemogenesis, including additional genetic mutations [78,79] and/or alterations in the bone marrow microenvironment, as these have been found to contribute to myeloproliferative syndromes, myelodysplasia and secondary leukaemia, and juvenile myelomonocytic leukaemia [80,81,82]. Leukaemogenesis results in the generation of futile, aberrant cells or “blasts”, which accumulate and promote multidimensional damage to the bone marrow environment, with subsequent impairment of the normal, healthy haematopoiesis [83]. 

**Figure 3:**
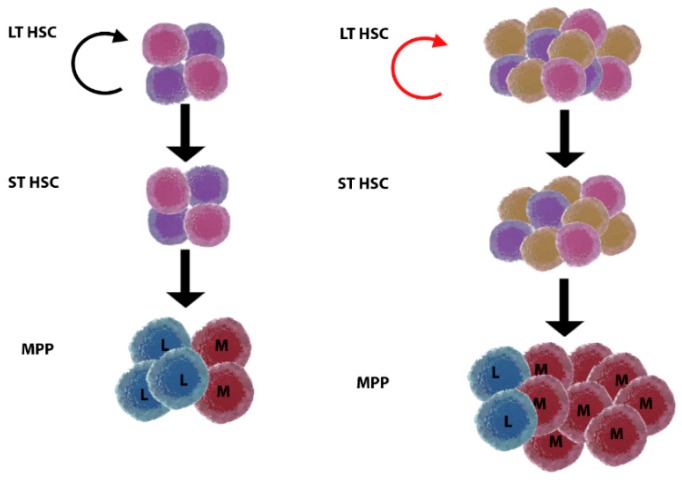
Model of age-related clonal haematopoiesis. Young (**left**) versus old (**right**) haematopoiesis. Long-term haematopoietic stem cells (LT HSC) are present in increased numbers but with reduced self-renewal ability (twisted arrow, red) in the elderly. Somatic mutations arise with ageing in LT HSC, and some of the clones are positively selected and expand, giving rise to age-related clonal haematopoiesis. This status may or not evolve to malignancy by acquisition of additional mutations. It is currently unknown how clonal haematopoiesis relates to inflammageing, why selection of clones originates myeloid skewing, and what is the role of the HSC niche. ST HSC, short-term haematopoietic stem cell; MPP, multipotent progenitor; L, lymphoid; M, myeloid.

Age-related clonal haematopoiesis is defined as the expansion of HSCs and progenitor clones, harboring specific, disruptive, and recurrent genetic variants, in individuals with no diagnosis of haematological malignancy [84] (Figure 3). Mutations in genes responsible for clonal expansion accumulate with ageing, resulting in widely asymptomatic clonal haematopoiesis. However, the same mutations are also associated with malignancies, such as those in the genes DNMT3A, JAK2, TET2, ASXL1, SF3B1, and TP53 [85,86,87]. Thus, age-related clonal haematopoiesis is a phenomenon that gives myeloid malignancies an evolutionary advantage in old patients, and it may be considered a preleukaemic condition [84,85]. Population screening shows that these mutations and the concurrent age-related haematopoietic clonal expansion were present in about 2% of individuals, or 5–6% of those above 70 years of age [87]. 

Furthermore, whereas the above mutations may be initiating events for haematological malignancies, the absence of detectable mutations in IDH1, RUNX1, NRAS, NPM1, and FLT3 suggests that these may be cooperating mutations in disease progression [87]. By the use of deep sequencing, a recent work has analysed genes that are recurrently mutated in AML to distinguish between individuals at risk of developing disease to those with benign age-related clonal haematopoiesis [88]. Peripheral blood cells from 95 individuals were obtained ~6.3 years before AML diagnosis, together with 414 unselected age- and gender-matched individuals. Preleukaemic cases had more mutations per sample, greater clonal expansion, and showed enrichment of mutations in specific genes, such as TP53 and U2AF1. Mutations in other genes, for example DNMT3A and TET2, seem to confer lower risk of malignant transformation [88]. However, preleukaemic HSCs, ancestral to the dominant leukaemic clone, are prevalent among patients with DNMT3A mutations and possibly also IDH2 mutations [89]. Ancestral clones may escape chemotherapy and persist at remission, representing a reservoir for relapse [89]. Thus, studies on age-related clonal haematopoiesis may hold the answers for early diagnosis and targeted therapies in patients with haematological malignancies. 

Interestingly, some studies have not found mutations in candidate driver genes, responsible for clonal expansion, in a significant fraction of individuals with clonal haematopoiesis [86]. Furthermore, an Icelandic population study revealed that clonal haematopoiesis is very common, almost inevitable, in the elderly, and driver mutations are not evident in most cases [90]. Although technical limitations may underlie these puzzling results, alternative explanations include clonally inherited epigenetic states [91], neutral genetic drifts operating on HSCs that would result in random clonal selection [92], and selective pressure by the bone marrow niche. In humans, a single case report gives a clue about the role of the bone marrow niche in disease development [93]. In this case, a patient of AML developed AML again after allogenic transplantation from his sibling. The second leukaemia was donor-derived, with mutations in IDH2 and DNMT3A detected only in the donor and not in the primary AML. The donor was monitored and never developed blood malignancy, in contrast to the recipient, who rapidly accumulated additional genetic hits. Several factors may have contributed to this outcome, such as the growth-promoting condition of the bone marrow after transplantation and a defective immune response [93]. However, it is interesting to see that external stimuli are decisive in clonal evolution and this points to the bone marrow niche as a key player for therapeutic strategies. More recently, ageing of the niche was found to influence clonality in haematopoiesis [94]. By generating retrovirally transduced SF91/IRES-eGFP dominant HSCs and progenitors of increased transplantation potential, transplantation of old recipient mice led to decreased HSC clonality and skewed differentiation towards myeloid lineage. This suggests that the aged niche promotes the transition to monoclonality, and thus facilitates leukaemia initiation [94]. It remains to be seen why clonal haematopoiesis arises; how it relates to the age-related changes in the haematopoietic system, in particular myeloid skewing and inflammageing; and what is the role of relevant HSC niche cellular components in these processes.

## 6. Conclusions

HSC ageing is characterised by reduced self-renewal, myeloid and platelet HSC skewing, and expanded clonal haematopoiesis that is considered a preleukaemic state. The underlying molecular mechanisms seem to be related to increased oxidative stress due to ROS accumulation and DNA damage, which are influenced by both cell- and cell non-autonomous mechanisms such as prolonged exposure to infections, inflammageing, immunosenescence, and age-related changes in the HSC niche. Thus, HSC ageing seems to be multifactorial and we are only beginning to connect all the dots. In mouse models, strategies such as restoring telomerase function and prolonged treatment with antioxidants or inhibitors of p38 MAPK have been successful at rejuvenating HSC function. Examples of cell non-autonomous therapies include the pharmacological targeting of insulin-like growth factor-1 in aged osteoblastic niche cells and treatment with adrenoreceptor beta 3 agonist. The latter not only significantly rejuvenates the in vivo function of aged HSCs, but also blocks disease progression in a model of age-related haematological malignancy. Thus, future work should accurately explore the sequence of events and players leading to HSC decay and transformation with ageing, aiming at developing integrative and efficient combinatorial strategies to slow down these processes.
